# Thymic Stromal Lymphopoietin (TSLP), Its Isoforms and the Interplay with the Epithelium in Allergy and Asthma

**DOI:** 10.3390/ijms241612725

**Published:** 2023-08-12

**Authors:** Sylwia Smolinska, Darío Antolín-Amérigo, Florin-Dan Popescu, Marek Jutel

**Affiliations:** 1 Department of Clinical Immunology, Wroclaw Medical University, 50-368 Wroclaw, Poland; sylwia.smolinska@umw.edu.pl; 2 Servicio de Alergia, Hospital Universitario Ramón y Cajal, Instituto Ramón y Cajal de Investigación Sanitaria (IRYCIS), 28034 Madrid, Spain; dario.antolin@gmail.com; 3 Department of Allergology “Nicolae Malaxa” Clinical Hospital, “Carol Davila” University of Medicine and Pharmacy, 022441 Bucharest, Romania; florindanpopescu@allergist.com; 4 “ALL-MED” Research Medical Institute, 53-201 Wroclaw, Poland

**Keywords:** thymic stromal lymphopoietin, epithelium, allergy, asthma

## Abstract

Thymic stromal lymphopoietin (TSLP) is a pleiotropic cytokine that has emerged as a critical player in the development and progression of allergy and asthma. It is primarily produced by epithelial cells and functions as a potent immune system activator. TSLP acts through interaction with its receptor complex, composed of the TSLP receptor (TSLPR) and interleukin-7 receptor alpha chain (IL-7Rα), activating downstream complex signalling pathways. The TSLP major isoform, known as long-form TSLP (lfTSLP), is upregulated in the airway epithelium of patients with allergic diseases. More research is warranted to explore the precise mechanisms by which short-form TSLP (sfTSLP) regulates immune responses. Understanding the dynamic interplay between TSLP and the dysfunctional epithelium provides insights into the mechanisms underlying allergy and asthma pathogenesis. Targeting TSLP represents an important therapeutic strategy, as it may upstream disrupt the inflammatory cascade and alleviate symptoms associated with allergic inflammation.

## 1. Introduction

The cytokine TSLP was initially identified in conditioned medium from a murine thymic stromal cell line with a medullary phenotype as a growth factor for B and T cells and co-stimulator for thymocyte proliferation, consequently suggesting its role as lymphopoietin. The human TSLP homolog was found using in silico methods, detecting the homology to mouse TSLP by a computational screen of the human genomic databases [1,2]. TSLP was categorized later on as a epithelial-derived crucial mediator of the type 2 immune responses and is nowadays considered as a cytokine with multiple functions and critical roles in diverse physiological and pathological conditions, including asthma, allergic diseases, chronic inflammatory diseases and cancer [1]. Some of these include regulation of immune responses, acting as an early alarm signal with significant roles in epithelial barrier function, dendritic cell activation, type 2 innate lymphoid cells (ILC2s) activation and survival, immune cell recruitment, induction of T2 responses and regulation of B cell function, explaining its involvement in tissue homeostasis, host defence and microbiome regulation, and in the pathophysiology of allergic and inflammatory diseases. TSLP is a cytokine mainly derived from epithelial cells, which occupies an upstream position in the asthma inflammatory cascade [1,2].

This review is intended to be an educational resource for researchers, clinicians and students interested in cytokine and epithelial cell biology, asthma and allergic diseases, by summarizing and discussing the current state of knowledge on the multifaceted roles of TSLP and its isoforms, and by focusing more on molecular approaches for a better understanding of the topic in its complexity and significance.

## 2. TSLP, Its Receptors and Molecular Interactions

TSLP is a four α-helical type I cytokine which plays critical roles in diverse physiological and pathological conditions [1,2].

Various stimuli, including mechanical injury, ligands for Toll-like receptors (TLR2, TLR3) and NOD2, pro-inflammatory cytokines and allergen proteases can induce the expression of the alarmin cytokine TSLP in epithelial cells [1,3,4].

In the lungs, TSLP production by epithelial cells with a rapid cellular release is also triggered by viral infections, including those with respiratory syncytial viruses, rhinoviruses, influenza viruses and metapneumoviruses, which incite other danger signals and exacerbate inflammation [3,5,6].

Moreover, when challenged by allergen molecules, barrier cells such as bronchial and intestinal epithelial cells can overproduce reactive oxygen species (ROS) by up-regulating nicotinamide adenine dinucleotide phosphate (NADPH) oxidase and activating the nuclear transcription factor NF-*κ*B signal pathway, leading to elevated cytokines including TSLP [7,8,9].

TSLP, mainly produced in response to pathogenic stimuli by lung and intestinal epithelial cells and skin keratinocytes, induces complex immune responses at barrier surface level by activation of dendritic cells (DCs), mast cells, eosinophils and lymphocytes into a type 2 polarizing phenotype [10,11]. Therefore, nowadays, TSLP is considered a master regulator of T2-driven inflammation [12].

TSLP activates intracellular signalling by forming a complex with its specific receptor, TSLPR (encoded by CRLF2) and IL-7Rα. The cognate receptor TSLPR allosterically triggers TSLP to potentiate the recruitment of the shared IL-7 receptor α-chain (IL-7Rα) by leveraging the cytokine molecule’s conformational heterogeneity, flexibility and electrostatics [13,14,15,16].

TSLP has a four-helix structure stabilized by three disulfide bridges, in which the four α-helices, designated αA to αD, are linked via a BC loop and two longer AB and CD loop regions. TSLP, positively charged, binds to TSLPR, negatively charged [15].

**Figure 1:**
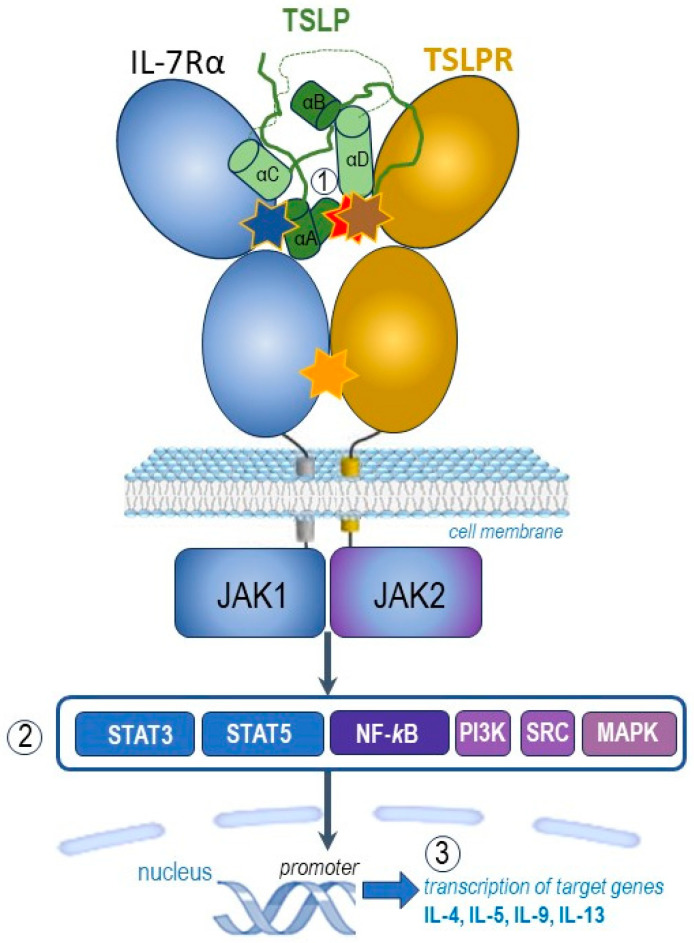
Graphic representation of the pro-inflammatory TSLP mediated complex and its intracellular signalling pathways (adapted after [1,13,14,15,16]). TSLP with an atypical open helical bundle core, wedges between TSLPR and IL-7Rα to mediate the T-shaped extracellular assembly. The brown star indicates site I (TSLP:TSLPR interface) with pronounced electrostatic complementarity, the blue star denotes site II (TSLP:IL-7Rα interface) and the orange one site III (IL-7Rα:TSLPR interface). TSLP, represented here in green, is the long-form TSLP (the common part with the short-form TSLP is presented in light green). The hidden red star highlights the binding region of tezepelumab overlapped by the binding region on TSLP on TSLPR. The parts noted 1, 2, and 3 of this figure represent complex molecular events at the receptor (1), transcription factors (2) and target genes (3) levels, as explained in the text.

TSLP engages its C-terminal short tail, C-terminal half of the helix αD and continuous 10 residues in the long AB loop region (Figure 1, part ①) to interact with cytokine-binding homology region of the receptor TSLPR (site I) with cooperative recruitment of IL-7Rα (site II), allowing the membrane-proximal parts of the two receptors to engage in heterotypic receptor–receptor interactions (site III). [1,15].

Following the capture and rearrangement of TSLP by TSLPR at the cellular surface, IL-7Rα is recruited to initiate intracellular pro-inflammatory JAK-STAT pathways (Figure 1, part ②). The JAK-STAT signalling pathway is an important chain of interactions between proteins in a cell, which involves Janus kinase (JAK) and signal transducer and activator of transcription (STAT) [1,12].

TSLP intracellular signalling cascades are initiated by JAK1/JAK2, molecules involved in phosphorylation of STAT3/5, but also include NF-*κ*B (nuclear factor *κ*B), MAPK (mitogen-activated protein kinases), PI3K (phosphoinositide 3 kinase), SRC (sarcoma tyrosine kinase) pathways. TSLP activates JAK1 (via IL-7Rα) and JAK2 (via TSLPR). JAK1 and JAK2 then activate signal transducer and activator of transcription 5 (STAT5) and, to a lesser extent, STAT3 and other transcription factors [1,13,14,15,16].

Finally, the transcription of target genes is promoted (Figure 1, part ③), including type 2 pro-inflammatory cytokines, such as IL-5 (key cytokine in eosinophilic inflammation), IL-9 (important cytokine in allergic inflammation), IL-4 and IL-13 (key cytokines in type 2 inflammation) [1,12].

Altogether, the binding of TSLP to its heterodimeric receptor complex TSLPR and IL-7Rα initiates intracellular activation of several downstream protein tyrosine kinases with phosphorylation of transcription factors which initiate the transcription of some target genes including those encoding pro-inflammatory cytokines involved in allergic and eosinophilic inflammation [12,16].

**Figure 2:**
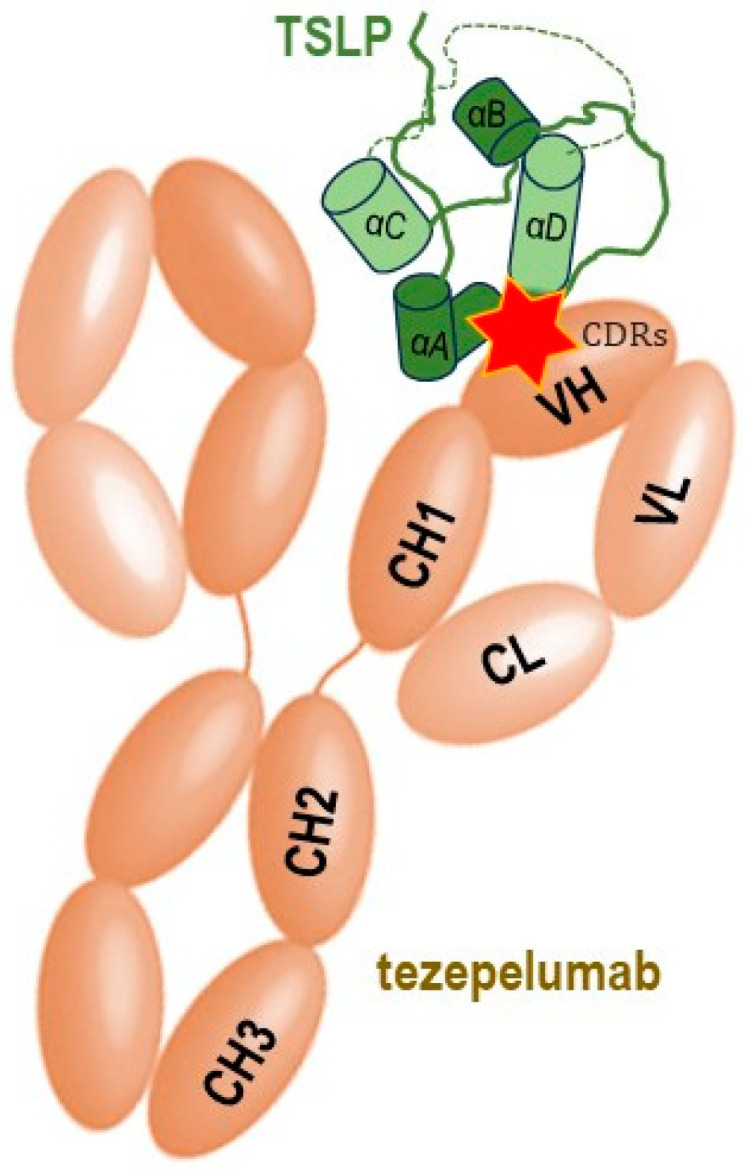
Schematic simplified representation of the molecular binding of the monoclonal antibody tezepelumab to the human TSLP with subsequent blocking of its interaction with the heterodimeric TSLP receptor, thus impeding the formation of the TSLPR: TSLP: IL-7Rα ternary complex on effector cells. The CDRs of the VH of tezepelumab target TSLP at the C-terminal region of helix αD and AB loop region (marked by red star), while the VL does not interact with TSLP (adapted after [15]). TSLP represented here in green is the long-form TSLP (the common part with the short-form TSLP is highlighted in light green).

TSLP engages the C-terminal part of the helix αD (residues 142–152), the C-terminal tail extending from αD (residues 153–158), with an important triplet of arginine residues, and a continuous sequence of 10 residues in the AB loop region (residues 60–69) to interact with a complementary epitope shaped at the cytokine-binding homology region of the receptor TSLPR (Figure 2). Key interactions between the C-terminal helix of TSLP and the binding groove of TSLPR were depicted. The strong binding is mediated by a network of hydrophobic interactions and hydrogen bonds between TSLP Leu156 and TSLPR Leu39 [12,15]. In addition, the AB loop may act as a link between the two receptor binding regions on TSLPR (sites I and II), balanced to dispatch the binding event to TSLPR at site I to prime TSLP for the cooperative recruitment of IL-7Rα at site II. The AB loop provides a physical link to αA, which is central to defining site II. Human TSLP-Leu45 interacts with both TSLPR and IL-7Rα [15].

Tezepelumab, a fully human monoclonal IgG2λ antibody currently indicated for the treatment of severe asthma, specifically binds human TSLP at the level of its ligation site for TSLPR, thereby impeding the TSLP-TSLPR interaction [16]. The complementarity determining regions of the VH (variable heavy chain domain) of tezepelumab target TSLP at the C-terminal region of helix D and AB loop region, while the VL (variable light chain fragment) does not interact with TSLP at all. Tezepelumab competes against a critical part of the TSLPR binding region on TSLP but remains completely clear of the IL-7Rα binding region on the other side of the TSLP helical bundle [15].

## 3. TSLP Isoforms

In humans, there are two TSLP variants: long-form TSLP (lfTSLP or TSLP variant 1) and short-form TSLP (sfTSLP or TSLP variant 2), their genes consisting of four and two exons, respectively, and are derived from the activity of two promoter regions [17]. The distinct regulation of sfTSLP and lfTSLP suggests different functions, including specific immunological roles [1,18].

These two TSLP isoforms have been identified initially in human bronchial epithelial cells, the lfTSLP, upregulated by a TLR3 ligand (poly I:C), and sfTSL, constitutively expressed in normal tissues [19]. The former is also acknowledged as TSLP and is the molecule that corresponds to mouse TSLP. Later research confirmed the existence of these two TSLP variants in human tissues: the isoform expressed in a steady state as sfTSLP, which plays a homeostatic role, and the isoform upregulated in inflammatory conditions, lfTSLP. In addition, in pathological conditions, TSLP can be cleaved by several endogenous proteases [20].

### 3.1. The Long Isoform of TSLP

Among the two TSLP isoforms, the lfTSLP has been more extensively studied, and it is recognized as a critical regulator of immune responses, particularly in inflammation and allergic reactions. The human TSLP gene is located on chromosome 5q22.1 near the atopic cytokine cluster. lfTSLP is closely related to IL-7, with which it shares a biological profile that is similar, but not identical. It binds to the previously described heterodimeric receptor complex consisting of IL-7Rα and TSLPR. TSLP and IL-7 are considered paralogues, likely having derived from a gene duplication event [1,2,20].

**Figure 3:**
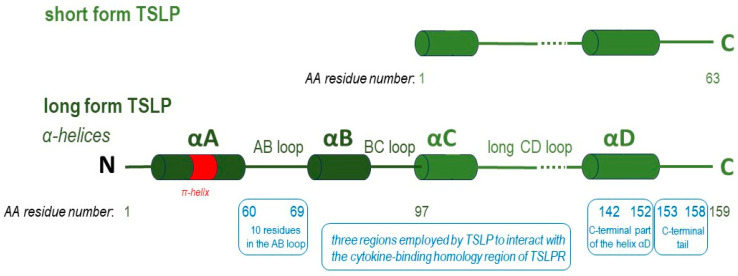
Long form TSLP (lfTSLP), consisting of 159 amino acids (AA), has four α-helices (αA, αB, αC, αD) linked by a BC loop and two longer AB and CD loop regions. Short form TSLP is 63 AA residues in length and covers the C-terminal half of human TSLP (residues 97–159) (adapted after [15]). The regions employed by TSLP to interact with the cytokine-binding homology region of TSLPR are marked in boxes in blue. Tezepelumab targets TSLP at the C-terminal region of helix D and AB loop region. The plasticity of the π-helical turn in αA has an critical functional role in the priming of TSLP by TSLPR to enable high-affinity binding by IL-7Rα; therefore, IL-7Rα cannot be recruited to the TSLP:tezepelumab complex.

The two TSLP isoforms are not the product of alternative splicing of the same transcript, but are instead controlled by two different promoter regions [17]. The lfTSLP has is a sequence of 159 amino acids (AA), while the sfTSLP has a sequence that is identical in the C-terminal region of the long isoform and consists of 63 AA [12,21]. A comparison of the structures of the two TSLP isoforms is presented in Figure 3.

The functional activity of lfTSLP is regulated by post-translational alterations. Proprotein convertase enzymes can truncate it between residues 130 and 131 to generate a heterodimeric short-lived metabolite, TSLP (29–130 + 131–159), which powerfully activates myeloid DCs and group 2 innate lymphoid cells (ILCs) compared to mature lfTSLP, thus driving type 2 inflammation. Carboxypeptidase N (CPN) then digests six AA from the C-terminus of the lfTSLP to produce a more stable dimerized form, TSLP (29–124 + 131–159). These truncations are primate specific and homeostatic. [12,20,22].

lfTSLP matches to the commercially available recombinant TSLP produced by prokaryotic cells. sfTSLP overlaps the lfTSLP in its terminal region, and the majority of the available anti-TSLP antibodies for different scientific immunoassays used to detect TSLP do not distinguish between the two isoforms. Therefore, a quantitative polymerase chain reaction using specific primers is the only way to determine the two TSLP variants at the molecular level. Several studies have examined the expression and functions of the two TSLP variants in different human diseases, but additional research is needed [20].

The lfTSLP isoform is primarily produced by epithelial cells, especially in tissues like the lung, gastrointestinal tract and the skin. DCs, basophils, mast cells, keratinocytes and fibroblasts also produce lfTSLP following stimulation [1,20].

The functional receptor for lfTSLP is expressed on many cells, including DCs, T and B cells, natural killer T cells (NKT), eosinophils, basophils and epithelial cells. Activation of the TSLPR induces multiple transcription factors including those of the JAK/STAT intracellular pathways, and thus lfTSLP impacts many immune responses and is associated with several immune pathologies, such as allergic diseases and intestinal inflammation. lfTSLP-stimulated DCs are involved in the inflammatory cells production of T2 cytokines such as IL-5, IL-4 and IL-13. This inflammatory type 2 phenotype is induced by upregulating OX-40 ligand expression on lfTSLP-treated DCs. Therefore, in respiratory allergy, exposure to lfTSLP of DCs potently augments allergen-specific TH2 responses. In atopic dermatitis (AD), lfTSLP is highly expressed in acute and chronic lesions but it is not detectable in non-lesional skin [10,20,23,24].

lfTSLP is induced at sites of inflammation having important proinflammatory actions. Thus, it can be vastly upregulated in inflammatory diseases, including asthma and AD. The expression of lfTSLP is triggered by TLR ligands, including poly I:C, FSL-1, and flagellin, and proinflammatory cytokines, such as IL-4, IL-13 and tumour necrosis factor-α (TNF-α) [17,19,25,26,27,28,29]. TLR3, TLR2 and TLR6 ligands induce lfTSLP in human keratinocytes in the presence of proinflammatory cytokines such as IL-4, IL-13 and TNF-α [25], and activation of human intestinal epithelial cells with cytokines such as IL-1 and TNF-α upregulates lfTSLP [30]. Moreover, TNF-α activation of human lung fibroblasts upregulates lfTSLP [26] and respiratory viruses, such as metapneumoviruses, strongly induces the expression of the lfTSLP isoform in human airway epithelial cells and in pulmonary fibroblasts [18].

Two single nucleotide polymorphisms (SNPs) in the TSLP promoter, rs2289276 and rs2289278, confer enhanced binding by the AP1 transcription factor and augmented lfTSLP production, explaining why these SNPs are associated with higher incidence of pediatric atopic disease and adult asthma [31].

lfTSLP damages airway barrier function in a murine model of asthma, suggesting its important pathogenesis contribution in the disease. Particularly, lfTSLP contributes to house dust mite (HDM)-induced bronchial epithelial dysfunction and the chromobox CBX4 regulates lfTSLP-mediated airway inflammation through SUMOylation (small ubiquitin-like modifier) in HDM-induced asthma [32]. In an HDM-stimulated airway smooth muscle cells as an in vitro model and in a murine HDM-induced asthma as an in vivo model, lfTSLP was revealed to induce autophagy-mediated asthmatic airway inflammation and remodelling [33].

The pathology-dependent pattern of expression of the lfTSLP compared with sfTSLP is presented in Table 1.

Interestingly, lfTSLP exerts antimicrobial activities and this is related to the last 34 AA of the cytokine. This could mean that the dysbiosis observed in many barrier surface pathologies could impact the homeostatic balanced expression of TSLP. Some highly immunogenic bacterial strains, such as adherent-invasive *Escherichia coli*, upregulate lfTSLP, whereas the opposite occurs after challenge with a commensal strain [33].

Additional research into lfTSLP’s precise mechanisms of action and its interactions with other cytokines and cells will offer a better and more detailed understanding of immune regulation and inflammation processes along with improved approaches for therapeutic interventions. The emergence of lfTSLP as a central orchestrator of type 2 responses capable of initiation of inflammation and allergy has preferentially positioned a new therapeutic targeting of the TSLP-mediated signalling in asthma and atopic diseases [15].

### 3.2. The Short Form TSLP

Compared to lfTSLP, sfTSLP has been less studied in humans, and its detailed physiological roles are still unknown. However, emerging evidence suggests that sfTSLP might play a unique position as a significant inflammation regulator. The sfTSLP mRNA seems human specific, as there are no published data of a similar variant in other species [20]. Transcription of sfTSLP initiates from a promoter in intron 2 and is thus truncated at the amino terminus. This short isoform 63 AA lacks the amino-terminal domain of the long one but shares the same AA sequence at the carboxy-terminal domain [12,19].

The sfTSLP is not upregulated by inflammation and plays a homeostatic role being constitutively expressed in a steady state in different tissues. Since it has homeostatic and anti-inflammatory effects, this was considered when designing targeted pharmacological strategies. Ideally, therapeutical monoclonal antibodies produced to neutralize TSLP should not interact nor hamper the known sfTSLP homeostatic functions. [1,20,34].

sfTSLP has been reported to be expressed in several types of cells, including T cells and mast cells. sfTSLP mRNA is also constitutively expressed in human epithelial cells, lung macrophages and lung fibroblasts. In addition, it is expressed in the skin and oral mucosa keratinocytes and in salivary glands, at the mRNA and protein level. Distinctive roles were suggested, the sfTSLP appearing to have the opposite effect to lfTSLP in immune modulation. Therefore, sfTSLP is considered to act as an inflammation suppressor with anti-inflammatory functions. This short TSLP isoform may counterbalance the effects of the long one, mitigating excessive immune responses and inflammation [1,17,21,35].

Administration of sfTSLP decreases airway hyperreactivity and ameliorated inflammation in an HDM-induced murine model of asthma [28,31]. sfTSLP treatment in vivo reverses HDM-mediated activation of inflammation and airway remodelling [33].

A specific receptor for sfTSLP has yet to be discovered. The striking difference between the two TSLP isoforms is that sfTSLP does not bind to the TSLPR and it cannot block the binding of lfTSLP to this receptor. The short isoform induces phosphorylation of p38 mitogen-activated protein kinase (MAPK) p38-α and extracellular signal-regulated kinase ERK 1/2 but has no significant effect on STAT5 [35].

sfTSLP exhibits potent antimicrobial activity, exceeding that of many known antimicrobial peptides (AMPs). As sfTSLP can be downregulated by inflammation, this might contribute to an aggravation of local infections. In healthy barrier surfaces such as skin and intestine, sfTSLP is the main isoform detected; therefore, it is expected to play an important local role against infection and in inflammation regulation. Vitamin D upregulates only sfTSLP in epithelial cells, a putative vitamin D receptor binding site being identified in intron 3 of the TSLP gene [33,35].

The expression of sfTSLP in the skin, oral mucosa, salivary glands and the intestine is part of the defence barrier that helps in controlling both commensal and pathogenic microbes [35]. For instance, a highly immunogenic *E. coli* strain downregulates the sfTSLP, whereas the opposite happens after challenge with a commensal strain [33].

Furthermore, it is important to underline that the sfTSLP is not only necessary for homeostasis, but it is also involved in carcinogenesis [1,29,36]. In human lung cancer, sfTSLP mRNA is significantly more expressed in the intratumoural tissue compared to the peritumoural area [37]. In ovarian, endometrial and cervical cancers, sfTSLP is mainly expressed and promotes tumour growth probably via the downregulation of the Ephrin-B2 (EFNB2) protein [38].

## 4. TSLP as a Critical Cytokine in Epithelium Interactions and T2 Responses

The dysfunctional airway epithelium and its derived cytokine TSLP represent a very dynamic upstream level within the multifaceted interplay of cellular and molecular complex pathways leading to airway inflammation, either type 2 one, including allergic and non-allergic asthma phenotypes, mostly outlined by eosinophilic inflammation, or T2-low one, featured by neutrophilic or paucigranulocytic patterns [16].

Exposure of the respiratory epithelium to allergens, viruses, bacteria, pollutants or mechanical injuries as environmental stimuli can promote loss of integrity as well as anchorage of the airway epithelium, causing the release of the alarmin cytokines represented by IL-25, IL-33 and TSLP [39]. Alarmins can be produced by healthy activated immune cells and secreted through the endoplasmic reticulum-Golgi apparatus or nonclassical pathways, such as nonprogrammed cell death. They promote the adaptive immune system directly or indirectly via the recruitment and activation of antigen-presenting cells (APCs), such as DCs and ILC2, and they can support homeostatic functions such as tissue repair [40,41,42,43,44]. Alarmins play key roles in asthma, in driving type 2-high and to a lesser extent type 2-low responses. In addition, studies targeting each of these three alarmins in allergen-challenged mice revealed reduced chronicity of type-2 driven processes [45].

**Figure 4:**
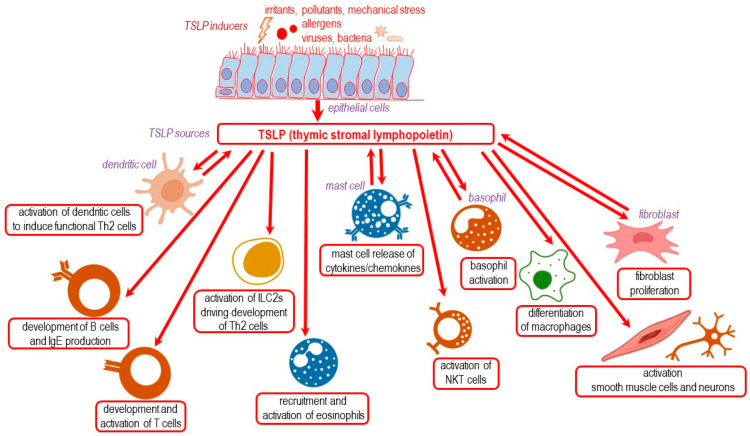
TSLP: environmental inducers, cellular sources and its variety of targets and actions (adapted after [1,46,47]).

The cellular sources of TSLP are in fact those presented as sources for lfTSLP, and include mainly epithelial cells, along with DCs, basophils, mast cells and fibroblasts. TSLP exhibits diverse effects on multiple cell types [1,46,47], including B and T cells, ILC2s, eosinophils, NKT cells, macrophages, smooth muscle cells and nerve cells. Additionally, TSLP influences the activity of DCs, mast cells and basophils. It mediates activation, proliferation and survival of some of these cells and links innate and adaptative immune responses (Figure 4).

TSLP promotes allergic responses by acting on DCs and inducing their expression of OX40 ligand (OX40L), CD80 and CD86, promoting the differentiation of naive CD4+ T cells into pro-inflammatory TH2 cells, which are capable of producing IL-5, IL-4, IL-13 and TNF-α [10,24]. Moreover, in a murine model with neonatal infection with respiratory syncytial virus (RSV) and reinfection, TSLP expression is associated with OX40L expression, lung DC migration and TH2 cell polarization, leading to allergic responses later in life [48,49]. Subsequently, TSLP-activated DCs also stimulate naive CD4+ T cells to differentiate into T follicular helper cells (defined by expression of IL-21, CXCR5, CXCL13 and BCL6), which can induce IgG and IgE secretion by memory B cells, linking TSLP to IgE production in allergy [50].

TSLP also promotes the release of T2 cytokines and chemokines by eosinophils, mast cells and macrophages [51,52,53]. Although the importance of TSLP in basophil responses is still not completely clear, a DC-T cell-basophil cascade has been involved in TSLP-driven type 2 immunity, through which DCs stimulated by TSLP prime CD4+ T cells via OX40L to induce IL-3, which then contributes to the recruitment of basophils and IL-4 production [54]. DCs themselves are known to produce TSLP upon TLR stimulation, suggesting that TSLP may also amplify the TH2 cell responses in an autocrine manner [55]. Besides the indirect effects of TSLP on CD4+ T cells, this alarmin also acts directly on CD4+ T cells [17,56] and is required for their full proliferation in response to antigen as well as for the generation of memory TH2 cells and recall responses. While DCs remain the most important target cell type for TSLP, other TSLPR-expressing cells also contribute to T2 cytokine-mediated allergic responses initiated by this alarmin. For instance, NKT cells expressing TSLP heterodimers are directly activated by TSLP to produce IL-13 [57].

Eosinophils are significantly affected by TSLP, since this alarmin delays eosinophil apoptosis, up regulates the expression of adhesion molecule ICAM-1 and CD18, enhances eosinophil adhesion onto fibronectin and induces the release of inflammatory cytokine IL-6 and several chemokines such as CXCL8, CXCL1 and CCL2. TSLP regulates these effects through the activation of p38 MAPK, ERK and NF-*k*B pathways, but not STAT5 and STAT3, which are usually activated in other effector cells upon TSLP stimulation. Moreover, TSLP also induces the release of IL-6 and the chemokines CXCL1, CXCL8 and CCL2 from eosinophils, as well as promoting the migration of neutrophils and non-hematopoietic cells [39,58].

The underlying mechanisms of epithelial cell interplay and disruption may be related to the differential capacity of inducing alarmins by allergen molecules with particular enzymatic and/or ligand-binding activity. HDM, Alternaria and cockroach allergen-derived proteases can induce TSLP, although experimental studies did not address specifically lfTSLP [39]. Protease allergens alter the integrity of the epithelial barrier and act as initiators/regulators of allergic inflammation. Those identified as stimulators of innate immune pathways include: papain-like cysteine proteases from HDMs (Der p 1, Der f 1) and ragweed pollen (Amb a 11), trypsin-like serine proteases from HDMs (Der p 3, Der f 3, Tyr p 3) and cockroaches (Per a 10), HDMs collagenase-like serin proteases (Der p 9) and fungal subtilisin-like serine proteases (Asp f 13, Pen ch 13) [59].

Epithelial injuries mediated by such proteases together with their sensing by protease-activated receptors (PARs) elicit strong inflammatory responses inducing the release of TSLP and other pro-T2 cytokines (IL-6, IL-25, IL-1β) and danger-associated molecular patterns (IL-33, ATP, uric acid). Importantly, Der p 1 degrades occludin and claudin proteins in tight junctions (TJ), while Asp f 13 also degrades E-cadherin in adherens junctions (AJ). Moreover, PARs, widely expressed in the airway epithelial cells, have been shown to be activated by protease allergens. Der f 3 activates PAR1, Der p 3, Der f 3 and Der p 9 activate PAR2 and Der p 3 can activate PAR4. Pen c 13 can also activate PAR1. In addition, Der p 1 induces thrombin-dependent activation of PAR1 and PAR4 by its capacity to mature prothrombin into thrombin, while a new fungal allergen epithelial-sensing mechanism converges on the caspase 8-ripoptosome activation and IL-33 maturation as contributors to type 2 innate immune responses. The nonselective ion channel TRPV1 (transient receptor potential vanilloid 1) is also important in the airway epithelial secretion of IL-33 in response to airborne protease allergens. Last but not least, proteolytic cleavage of fibrinogen can trigger TLR4 signalling, and cleavage of various cell surface receptors further optimize the TH2 polarization. Der p 1 and Per a 10 selectively shed cell surface receptors such as the low-affinity IgE Fc receptor CD23 (FcεRII) on B cells and the IL-2 receptor CD25 on both CD4 and CD8 T cells, while Der p 1 also sheds CD40 directly from DCs surface and cleaves DC-SIGN (CD209), a C-type lectin receptor primarily expressed on target cells [59,60]. C-type lectins have been involved in the recognition of HDM (Dectin-1, Dectin-2, DC-SIGN, Mannose Receptor) and recently the LMAN1 cargo receptor for a select set of glycoproteins was identified as a receptor for HDM allergens, their recognition being dependent on glycosylation (likely mannosylation). This implies that LMAN1 may serve as a general sensor of other mannosylated allergens such as peanut Ara h 1, cockroach Bla g 2 and dog Can f 1 [61].

## 5. TSLP in Asthma, Nasal Polyposis, Allergic Rhinitis and Ocular Allergy

Asthma risk is influenced by the combined effects of TSLP genotype and TSLP expression in the nasal epithelium rather than circulating TSLP. The joint effect of TSLP rs2289277 and rs11466750 genotypes and TSLP (sfTSLP and lfTSLP) expression in nasal epithelial cells is associated with increased childhood asthma [62]. Such localized expression of these isoforms indicates important local microenvironment homeostatic and proinflammatory roles rather than systemic circulation.

As previously mentioned, the central role of TSLP in airway inflammation is to activate CD11c+ myeloid DCs to express OX40L, which then primes naïve CD4+ T cells to differentiate into a pro-inflammatory T2 phenotype expressing IL-5, IL-4, IL-13 and TNF-α. When activated, CD4+ T cells are maintained and expanded through TSLP activated DCs expressing the PGD2 receptor CRTH2, which contribute to the proliferation of TH2 memory cells [10,24,63,64,65,66]. T regulatory cells interacting with TSLP-activated DCs (via OX40L) switch from an IL-10 producing regulatory subtype into a proinflammatory TNF-α producing one [24]. TSLP-activated DCs promotes tissue damage by activating naïve CD8+ T cells and induction of cytotoxic effector cells that can produce IL-5, IL-13 and a large amount of IFN-γ [1,10,24,65,66].

In severe asthma, TSLP is a key epithelial cytokine positioned at the top of the inflammatory cascade. For some patients with severe asthma, the epithelium is a starting point for an overactive immune response to various insults including allergens, pollutants/irritants, viruses, bacteria and other external stimuli. The heterogenous response to asthma treatment is likely directly related to differences in patterns of airway inflammation. The overexpression of TSLP can result in pathologic inflammation pathways driving various inflammatory phenotypes such as allergic, eosinophilic, neutrophilic and paucigranulocytic, which cause symptoms and asthma exacerbations [16,64,67,68].

In allergic asthma, by activation of DCs, TSLP promotes the differentiation of TH2 lymphocytes secreting T2 cytokines targeting B cells, eosinophils, mast cells and airway smooth muscle cells. In non-allergic eosinophilic asthma, TSLP stimulates ILC2 to release IL-5 and IL-13, while in neutrophilic asthma, TSLP induces DCs to induce neutrophil-activating TH17 lymphocytes. Moreover, in paucigranulocytic asthma, TSLP mediates multifaceted cross talks between inflammatory cells, such as mast cells and airway structural cells, such as epithelial cells, smooth muscle cells and fibroblasts [16]. Importantly, TSLP contributes to the pathogenesis of corticosteroid-resistant airway inflammation by Bcl-xL expression via ILC2s [69,70]. TSLP may also be considered a connection between the innate immune response to respiratory viral infections and the type-2 adaptive immune response in asthma [71]. Although anti-TSLP biologic treatment reduces exacerbations in severe asthmatics and it is assumed to suppress viral-induced ILC2-mediated eosinophilic inflammation as proven in a murine model [6], the definite effect of such a treatment on viral-induced innate responses and ILC2 activation in patients with asthma needs further assessment [72]. Moreover, TSLP could be a cytokine of interest also in non-severe asthma according to a recent study assessing plasma TSLP in asthmatic adults [73].

Human clinical trials of TSLP inhibition with the biologic tezepelumab completed to date have produced favourable results in patients with different asthma phenotypes, who experienced substantial improvements in lung function, symptom control and quality of life as well as reductions in exacerbation rates; this supported its recent approval for clinical practice in adults and adolescents with severe asthma [68].

Examination of nasal polyps from individuals with nonsteroidal anti-inflammatory drug (NSAID) aspirin-exacerbated respiratory disease (AERD)—NERD and those with chronic rhinosinusitis (CRS) without NERD/AERD showed that TSLP mRNA expression was significantly increased in NERD/AERD [74,75,76,77]. Cystatin SN (CST1), a type 2 cystatin subfamily member, is highly expressed in nasal polyps from patients with intractable chronic rhinosinusitis with nasal polyps [78]. CST1 expression can be induced by TSLP and can also stimulate TSLP expression in the presence of IL-4. Ongoing research may confirm the role of therapeutic targeting of TSLP with monoclonal antibodies such as tezepelumab in chronic rhinosinusitis with nasal polyps [79].

In allergic rhinitis, TSLP plays a central role in the initiation and persistence of allergic responses. The histamine H4 receptor regulates TH2-cytokine profile through TSLP in nasal allergy and the ubiquitin-specific peptidase 25 (USP25) downregulation involved in inflammation and the immune responses enhances the TSLP signalling in the nasal epithelium via decreased expression of TNF receptor-associated factor 3 (TRAF3), thereby exacerbating inflammation in allergic rhinitis [80]. Recently it was reported that inhibition of TSLP using tezepelumab increases the efficacy of subcutaneous allergen immunotherapy in patients with allergic rhinitis to cats and may promote tolerance after a one-year course of treatment [81].

In addition, TSLP may be an important factor involved to the development and progression of ocular allergy. TSLP is significantly expressed in the epithelial cells and some inflammatory cells of giant papillae from vernal keratoconjunctivitis (VKC) a rare, severe phenotype of chronic ocular allergy. Using immunohistochemical staining, OX40L and CD11c immunoreactive cells largely infiltrates such giant papillae. TSLP activates DCs to prime CD4+ T cells to differentiate into TH2 type and triggers T2-dominant allergic inflammation via TSLP/OX40L/OX40 signalling as part of pathogenesis of VKC [82].

## 6. TSLP in Urticaria, Atopic Dermatitis and Food Allergy

Because it is known that TSLP induces mast cell development, prevents apoptosis in skin mast cells and it is markedly upregulated in the wheals of patients with chronic spontaneous urticaria, the anti-TSLP monoclonal antibody tezepelumab is undergoing clinical trial assessments for this disease [83].

In AD, the overexpression of TSLP in keratinocytes is involved in type 2 inflammatory responses. DNA demethylation of a specific region of the TSLP promoter increases the expression of TSLP in skin lesions of patients with AD and reduces the expression of filaggrin, a structural protein for which the loss-of-function mutations are associated with epidermal barrier defects and more severe AD. [84,85]. Genetic variants of TSLP affect the severity and persistence of this disease. For example, rs1898671 homozygotes are associated with a reduced risk of AD in children, while the association of rs2289278 with AD is stronger in children with allergic sensitization. Furthermore, TSLP polymorphism also increased the risk of asthma in children with AD [86,87]. TSLP is highly expressed by keratinocytes in both acute or chronic AD lesions and it interacts with multiple immune cells in the pathogenesis of AD, being correlated with AD deterioration. TSLP is not found in nonlesional skin in AD patients. The stimulation of H4 receptors up-regulates the TSLP release from keratinocytes. By acting directly on sensory neurons TSLP additionally triggers itching [76,77,88]. The keratinocyte-derived cytokine TSLP is a key player in the early stages of inflammation. It potently activates DCs capable of priming naïve CD4+ T cells to differentiate into Th2 cells. There is also a direct induction of T cell migration by TSLP in the absence of DCs. TSLP downregulates FLG expression in human skin by STAT3 and/or ERK-dependent pathways. Serum TSLP levels in adults and children with AD are significantly higher compared to those in healthy individuals [89]. Therefore, TSLP may be an important target for AD treatment. It was reported that patients with IgE-high DPP-4–high, or periostin-low moderate to severe AD may have a better clinical response to tezepelumab, but additional clinical studies are required to confirm such findings [90].

TSLP is not found in the lesions of nickel-induced allergy contact dermatitis [88].

Eosinophilic esophagitis (EoE) is associated with polymorphisms in the gene encoding TSLP. A mouse model of EoE-like disease was developed independently of IgE, but dependent on TSLP and basophils. Therapeutic monoclonal antibody-mediated TSLP neutralization or basophil depletion ameliorated the established EoE-like disease in animals. In human subjects with EoE, elevated TSLP expression and exaggerated basophil responses in oesophageal biopsies were noticed, and a gain-of-function TSLP polymorphism was associated with increased basophil responses in such patients. Therefore, it was suggested that the TSLP-basophil axis contributes to the pathogenesis of EoE and has the potential to be therapeutically targeted [91].

In food allergy, alarmin-targeted monoclonal antibodies may be potentially useful in selected patients, but there is not yet enough certainty to support them. Etokimab, an antagonist of IL-33, has been recently studied for IgE-mediated peanut allergy, while no clinical trials with tezepelumab in food allergy have been reported. Large, randomised trials are needed to assess the safety, efficacy, posology and the most suitable candidates for such treatments [92,93]. The rationale for such studies derived initially from the functional role of TLSP in food allergy revealed in murine model studies [94]. In mice, epicutaneously sensitized with ovalbumine or peanut on AD-like skin lesion followed by intragastric allergen-specific challenge, expanded TSLP-elicited basophils in the skin were observed [90]. Epicutaneous sensitization to food allergen and development of food allergy may occur without skin inflammation and is partly mediated by TSLP, suggesting that its therapeutic targeting may be beneficial in food allergy early in life in at-risk infants [83]. Additional murine studies noticed that TSLP is an essential, but not exclusive, mediator in eliciting food allergy [95]. TSLP has a critical role in TH2 responses during the sensitization phase of food allergy, while IL-33 is important in inducing IgE-dependent anaphylaxis [96]. The role of TSLP in the pathophysiology of IgE-mediated food allergy is also discussed in human studies [97]. In adult patients with shrimp allergy, TSLP serum levels are elevated [98], while it was suggested its role also in the induction of food tolerance in patients with cows’ milk allergy [99].

Finally, growing evidence indicates the significant role of commensal microbiota in susceptibility to food allergies [100]. Exposure to certain pathogen-associated molecular patterns or injury to the epithelium, leading to the expression of TSLP, induces mucosal DCs to acquire a phenotype favouring TH2 cell priming when induced by the food antigens [101]. Recently, a critical peripheral tolerance axis between TSLP and DCs in the colon blocking CD4+ T cell activation against the commensal gut microbiome was discussed [102]. The oral microbiome of peanut-allergic individuals was characterized by reduced species in *Bacteroidales* (*Prevotella*), along with *Bacillales* and *Lactobacillales*, and oral *Prevotella* spp. abundance is correlated with decreased local secretion of TSLP [103].

## 7. Overview of the Pharmacological Anti-TSLP Strategies

Important lessons may be learned from animal studies using RNA sequencing and mouse models with deleted TSLP or TSLPR. Both murine and human TSLP exert their biological activities by binding to a high-affinity TSLPR complex, the heterodimer of TSLPR chain and IL7Rα [13,14], and even though the mouse and human sequence homology for both TSLP and its receptor is only about 40%, they have similar biological functions [63,104]. Experiments using RNA sequencing and transgenic mice suggested that TSLP undoubtedly contributes to TH2 responses [105] and TSLP receptor signalling is required to maintain ILC2s [106] with JAK-STAT as a TSLP signalling pathway [107]. Moreover, allergic inflammation in rat lungs and nasal epithelium is regulated by tissue-specific miRNA expression (hsa-miR-223-3p) involving the NF-*k*B signalling pathway [108].

Because TSLP is mainly an epithelium-derived alarmin, with a critical upstream role in the initiation of different immune responses [69], in transgenic mice with TSLP overexpression specifically in the lung or skin, the inflammatory response is accompanied by an increase in IgE and type 2 cytokine levels, which contributes to asthmatic or atopic dermatitis-like manifestations [109,110]. On the other hand, knocking out TSLPR and intratracheal administration of anti-TSLPR antibody suppresses the type 2 cytokine and IgE production in a mouse asthma model [111,112]. TSLP-deficient mice (TSLP^-/-^) have no apparent immune developmental deficiency [113] and TSLP deficiency decreases mast cell-mediated allergic reactions via STAT6 down-regulation [114]. In TSLPR-deficient mice (Tslpr^-/-^) it was revealed that TSLP activates different immune cells such as ILC2s, but also DCs and CD4 + T cells, in the framework of innate and adaptive type 2 inflammation [69]. In addition, it protects in a murine model of airway damage and inflammation via regulation of caspase-1 activity and apoptosis inhibition [115].

Levels of both TSLP mRNA and protein are increased within the airway epithelium and submucosa in asthma compared with healthy controls [64]. In a subset of patients with severe asthma, TSLP expression remains enhanced, independent of treatment with high-dose inhaled or oral corticosteroids [116]. Therefore, TSLP signalling blockade is a highly promising therapeutic approach for allergic diseases and asthma [117,118,119].

Several pharmacological strategies used for this purpose targeted TSLP or TSLPR as monoclonal antibodies or antagonists, and even fusion protein vaccines, for the treatment of inflammatory diseases [104]. Bispecific anti-TSLP/IL13 antibodies called Doppelmabs (bivalent) and Zweimabs (monovalent) were also assessed in initial research phases [120]. Humanized IgG1 monoclonal antibodies against TSLPR (RG7258, ASP7266) were also investigated in preclinical or phase I studies. Other approaches include TSLP cytokine traps, and small molecules targeting TSLP, such as baicalein [119,121]. Ecleralimab (CSJ117) is in undergoing development as an innovative dry powder inhaled anti-TSLP Fab antibody fragment that specifically targets the soluble TSLP, effectively preventing TSLP receptor activation [122,123].

Tezepelumab is the first-in-class fully human IgG2 monoclonal antibody against TSLP. It has been developed for the treatment of severe asthma, chronic obstructive pulmonary disease, chronic rhinosinusitis with nasal polyps, chronic spontaneous urticaria and eosinophilic oesophagitis [124]. This anti-TSLP biologic administered by subcutaneous injection was recently approved in clinical practice for the add-on maintenance treatment of adult and paediatric patients aged 12 years and older with severe asthma, irrespective of baseline biomarkers and regardless of the underlying endotype or phenotype [125,126]. Tezepelumab specifically binds human TSLP at the level of its ligation site for TSLPR, in that way preventing the TSLP-TSLPR interaction. By impending the binding of this relevant alarmin in the pathogenesis of asthma with its receptor, tezepelumab inhibits multiple downstream inflammatory pathways and reduces biomarkers and cytokines associated with inflammation including blood eosinophils, airway submucosal eosinophils, FeNO, IgE, IL-5 and IL-13 [16]. This type of biologic may have advantages by blocking such an upstream mediator with a potentially broader therapeutic impact compared with inhibiting downstream cytokines and/or their receptors [127].

## 8. Conclusions

TSLP is a pleiotropic cytokine that plays a pivotal role in the development and advancement of allergy and asthma. It is primarily produced by epithelial cells and serves as a potent immune system activator. TSLP exerts its effects by selectively binding to a high-affinity heteromeric complex composed of TSLPR and IL-7Rα, initiating downstream complex intracellular signalling pathways. Its isoforms exhibit distinct roles and functions, with lfTSLP as a potent inducer of immune responses and sfTSLP as a potential inflammation suppressor. Their biological functions and interactions could be a decisive factor in numerous conditions, such as allergy and asthma, offering valuable approaches for therapeutic development. A deeper understanding of the actions of TSLP isoforms and interplay with epithelial cells and immune cells is critical for developing safe and effective TSLP-targeted therapies in inflammatory disorders and beyond.

## Figures and Tables

**Table 1:** Comparative expression of TSLP isoforms in several inflammatory diseases (adapted after [34]).

Barrier Surface Disorder	Long TSLP	Short TSLP
Asthma	↑↑ upregulated	- unaffected
Atopic dermatitis	↑ upregulated	↓↓ downregulated
Ulcerative colitis	↑ upregulated	- unaffected
